# Overactive bladder syndrome and bladder wall thickness in patients with obstructive sleep apnea syndrome

**DOI:** 10.1590/S1677-5538.IBJU.2017.0253

**Published:** 2018

**Authors:** Zahide Yilmaz, Bekir Voyvoda, Pinar Bekdik Şirinocak

**Affiliations:** 1 Clinic of Neurology, Health Sciences University, Kocaeli Derince Training and Research Hospital, Turkey; 2 Clinic of Urology, Health Sciences University, Kocaeli Derince Training and Research Hospital, Turkey

**Keywords:** Urinary Bladder, Overactive, Urinary Bladder, Nocturia

## Abstract

**Objective:**

The main objective of the present study was to evaluate the presence of overactive bladder (OAB) syndrome, nocturia, urgency, and urge incontinence in patients with obstructive sleep apnea syndrome (OSAS), and measure bladder wall thickness (BWT) in these patients.

**Materials and Methods:**

The patient group was composed of 38 patients with OSAS. The control group was composed of 15 healthy individuals. All patients were evaluated using the Epworth Sleepiness Scale (ESS) and Overactive Bladder Symptom Score (OABSS). The bladder wall thickness was measured by transabdominal ultrasound (US). The presence of nocturia, urinary urgency, and urge incontinence were also evaluated.

**Results:**

The mean OABSS was significantly higher in the patient group compared with the control group (p=0.048). The minimum oxygen saturation (Min.SO_2_) of patients with urgency was found to be significantly lower (p=0.014). The time spent below 90% of oxygen saturation (SO_2_) was significantly longer in patients with urinary urgency (p=0.009). There was no difference in BWT measurements between the patient group and the control group. There was a significant relationship between BWT values and OABSS in patients with OSAS (p=0.002).

**Conclusion:**

The results of the present study suggest that OSAS is associated with OAB syndrome. As a key symptom of OAB, urgency correlates with hypoxia in cases with OSAS. Although the present study did not observe any difference in BWT measurements between the patients and the control group, there was a correlation between BWT measurements and OABSS in patients with OSAS.

## INTRODUCTION

Obstructive sleep apnea syndrome (OSAS) is a common disorder characterized by instability of the upper airway during sleep, resulting in reduction or elimination of the airflow, oxygen desaturation, and sleep disturbances (1). Approximately 4% of middle-aged men and 2% of middle--aged females are affected (1). Several studies have been conducted to evaluate possible association between OSAS and urological symptoms such as nocturia and erectile dysfunction (2, 3). However, the association of OSAS with overactive bladder (OAB) syndrome has been recently described (4).

Overactive bladder syndrome is defined as a symptom-based clinical diagnosis of urinary urgency with or without urgency incontinence (UI), usually with urinary frequency and nocturia, in the absence of infections or other evident pathological features (5). The key symptom of OAB is urgency and sudden, compelling desire to void that is difficult to defer (6). Several reasons have been suggested to be responsible for the development of OAB including morphological changes of the detrusor muscle (e.g., patchy denervation of detrusor muscle bundles), neurological changes (e.g., ischemic nerve damage), age-related causes of urinary dysfunction, and metabolic causes (e.g., disturbed serotonin metabolism) (6). In Europe, the prevalence of OAB is 4-15 % in men and 14-40 % in women (7).

Nocturia is the main symptom of lower urinary tract problems, which are often attributable to an underlying benign prostatic hyperplasia (BPH) in men. However, nocturia can represent several possible systematic health-related disorders, including OSAS. Definitions of nocturia have been previously published by the Standardization Committee of the International Continence Society (ICS) as the need to awake at night one or more times to void. Nocturia represents the number of voids recorded during a night's sleep (8).

As a diagnostic marker, potential use of bladder wall thickness (BWT) values in patients with OAB has been a current issue. In patients with OAB syndrome, BWT measurements are usually performed with transvaginal ultrasound (US) in women, and consistent results have been reported (9). Compared to transvaginal US, transabdominal US is more feasible, more cost-effective, and preferable in the ambulatory setting in daily urology practice (10). We did not find a study in the literature, which measured bladder wall thickening (BWT) in patients with OSAS. Therefore, the present study measured BWT using transabdominal US in patients with OSAS.

The aim of the present study was to evaluate the presence of OAB syndrome, nocturia, urgency, and urge incontinence in patients with mild, moderate and severe OSAS, and measure BWT in these patients.

## MATERIALS AND METHODS

### Study Population

After obtaining an informed consent from each participant, the patients who were diagnosed with OSAS using full-night polysomnography (PSG) at the sleep disorder laboratory of Kocaeli University, Health Sciences Institute, Derince Training and Research Hospital between December 2015 and December 2016 were screened. Patients with neurological disorders (e.g., multiple sclerosis, stroke, and dementia), metabolic disorders (e.g., diabetes mellitus), congestive heart failure, and those with a history of hypnotic, diuretic and anticholinergic drug use or who underwent urogenital operations (e.g., radical prostatectomy, transurethral resection) were excluded from the study. A total of 38 patients with OSAS were included in the study. The control group was composed of 15 individuals who were suspected of having a sleep disorder, but found to be normal on full-night PSG results. Exclusion criteria were also evaluated in the control group. The study protocol was approved by the local Ethics Committee. The study was conducted in accordance with the principles of the Declaration of Helsinki.

### Polysomnographic examination

A full-night PSG was performed in all patients and controls at the sleep disorder laboratory (Embla N 7000). The PSG examination included electroencephalogram, electro-oculogram, chin and leg electromyogram, electrocardiogram, snoring, thermistor, nasal pressure transducer, finger pulse oximetry, thoracic and abdominal respiratory movements, and body position. The scores were determined according to the 2010 American Academy of Sleep Medicine criteria. Patients with an apnea/hypopnea index (AHI) of ≥5/hour were considered to have OSAS. An AHI value between ≥5/hour and <15/hour was defined as mild; an AHI value ≥15/hour and less than 30/hour was defined as moderate; and an AHI value >30/hour was defined as severe OSAS.

Age, sex, body mass index (BMI), and Epworth Sleepiness Scale (ESS) scores of all patients were recorded. During PSG, the rate of sleep periods, AHI values, minimum oxygen saturation (Min.SO_2_), and the percentage of sleep time spent below 90% of oxygen saturation (time SO_2_<90%) were noted.

All patients were administered Turkish version of Overactive Bladder Symptom Score (OABSS-V8) and the IPSS-QoL; and BWT measurements were carried out under US guidance. In addition, the presence of nocturia, urgency, and UI was examined. The Beck Depression Inventory (BDI) and Beck Anxiety Inventory (BAI) were also administered.

The present study used the Turkish version of OABSS-V8. Tarcan et al. (11) analyzed the validity and reliability of the Turkish version of OAB--V8. At the end of this study, the Turkish version of OAB-V8 was found to be a valid, reliable, easily understood, and applicable test specific to the OAB syndrome. The cut-off value recommended for the total score in this study is 11. This value was considered as having the highest sensitivity (80%) and specificity (78%) in distinguishing patients from healthy individuals. A value above 11 was also considered to indicate the presence of OAB syndrome.

International Prostate Symptoms Score (IPSS-QoL) usually provides objective assessment of the questionnaire. IPSS-QoL was used only in men. This scale contains seven questions related to prostatic symptoms and one question related to quality of life. Using IPSS-QoL, values between 0 and 7 were considered to indicate mild obstruction, values between 8 and 19 to indicate moderate obstruction, and values between 20 and 35 indicate severe obstruction.

All patients underwent suprapubic transabdominal US (GE Logiq C2, 2010 India). The 4C-RC Convex ultrasound probe was used. BWT was measured with approximately 250-300cc of urine in the bladder (pre-voiding). Bladder measurement was made from the thickest point of the right or left wall.

#### Statistical analysis

The NCSS (Number Cruncher Statistical System) 2007 (Kaysville, Utah, USA) program was used for statistical analysis. In addition to the descriptive statistical methods (mean, standard deviation, median, first quartile, third quartile, frequency, ratio, minimum, maximum) used in the evaluation of the study data, Student's-t test was used for two-group comparisons of variables with normal distribution, and Mann-Whitney U-test was used for two-group comparisons of variables without normal distribution for the quantitative data. Kruskal-Wallis test was used to compare variables without normal distribution between more than two groups. Spearman's Correlation Analysis was used for evaluating the relationship between variables. Intragroup comparison of parameters without normal distribution was made using Wilcoxon Signed Ranks test. The Fisher-Freeman--Halton exact test, Fisher's exact test and the Yates’ Continuity Correction test (Yates’ correction Chi-square test) were used for the comparison of the qualitative data. Logistic regression analysis was performed in order to evaluate all factors affecting nocturia, urgency, and UI. Linear regression analysis was performed in order to evaluate all the factors affecting BWT and OABSS. A p value <0.05 was considered statistically significant.

## RESULTS

AHI, Min.SO_2_ and the time spent <90% oxygen saturation were found to be significantly lower in the patient group (p=0.001, p<0.01; p=0.001, p<0.01; p=0.001, p<0.01). However, no significant difference was found in the ESS scores between the two groups, although higher scores were observed in the patient group (Table-1).

**Table 1:** Evaluation of AHI, Min.SO_2_, time SO_2_<90%, and ESS scores of non-REM and REM stages according to the groups.

		Group		bp
		Patient (n=38)	Control (n=15)	
Non-REM stage 1 (%)	Min-Max (Median)	1.2-19.6 (5)	1.5-9.8 (2.7)	**0.018** *
	Mean±SD	6.46±4.40	3.91±2.79	
Non-REM stage 2 (%)	Min-Max (Median)	2.1-68.2 (9.8)	2-61.3 (10.2)	0.664
	Mean±SD	14.69±13.51	15.24±17.74	
Non-REM stage 3 (%)	Min-Max (Median)	20-88.5 (67)	13.7-83.5 (68.9)	0.992
	Mean±SD	65.56±15.05	62.75±19.77	
REM (%)	Min-Max (Median)	3.5-23.9 (14)	4.8-42 (18.6)	**0.026** *
	Mean±SD	13.32±5.23	19.14±8.78	
Min.SO_2_	Min-Max (Median)	50-94 (82.5)	86-96 (91)	**0.001** **
	Mean±SD	80.47±9.59	90.80±3.21	
Time SO_2_ <90 (%)	Min-Max (Median)	0-65.8 (0.9)	0-0.4 (0)	**0.001** **
	Mean±SD	8.16±16.38	0.05±0.12	
ESS	Min-Max (Median)	0-20 (12.5)	1-16 (7)	0.066
	Mean±SD	11.03±6.01	8.00±4.24	
AHI(/h)	Min-Max (Median)	0.8-4.8 (3.8)	5.5-97.1 (31.5)	**<0.001** **
	Mean±SD	3.04±1.39	39.06±26.98	

b Mann-Whitney U Test;

* p<0.05;

** p<0.01

**Abbreviations: Non-REM** = Non-Rapid Eye Movement; **REM** = Rapid Eye Movement; **Min.SO^2^** = Minimun oxygen saturation; **Time SO^2^ <90** = The time spent below 90% of oxygen saturation; **ESS** = Epworth Sleepiness Scale; **AHI** = Apne-Hypopne Index

We initially analyzed individual effects of age, gender, BMI and group on OABSS, and then performed a logistic regression analysis including all variables. The groups were different in terms of OABSS (p=0.048). The OABSS values of severe cases were significantly higher than the OABSS values of cases with no OSAS (p=0.049). In the model obtained as a result of the multivariate analysis (F=3.579, p=0.006), gender variable was found significant. It was found that the OABSS value increases to 5.584 if the case is female [Beta (95% CI): 5.584 (0.749, 10.419), p=0.025] (Table-2).

**Table 2:** Univariable and multivariable analysis of effects of age, gender, BMI and group variables on OABSS.

OABSS		Univariable analysis		Multivariable analysis	
		r	p	Beta	p
Age(year)		0.168	0.239	0.223	0.164
Gender, median (Q1, Q3)	Male	11.5 (7.5, 17)	0.155	-	-
	Female	17 (9, 27)		5.584	**0.025** *
BMI(kg/m^2^)		0.339	0.013*	0.298	0.116
Group, median (Q1, Q3)	Control	9 (7, 15)	**0.048** *	-	-
	Mild	13.5 (6, 23)		3.171	0.365
	Moderate	12 (4, 19)		-2.201	0.576
	Severe	17 (10, 27)		6.368	0.056

**Univariable analysis:** Spearman correlation was used for age and BMI, Mann-Whitney U test and Kruskal Wallis test were used for gender and group comparison, Multivariable analysis: Linear regression analysis.

* p<0.05

**Abbreviations: BMI** = Body Mass Index; **OABSS** = Overactive Bladder Symptom Score

Individual effects of age, gender, BMI and group on BWT were not significant. There was no significant difference in BWT measurements between the groups (Table-3).

**Table 3:** Univariable and multivariable analysis of effects of age, gender, BMI and group variables on BWT.

BWT		Univariable		Multivariable	
		r	p	F	p
Age(year)		0.187	0.188	1.608	0.168
Gender, median (Q1, Q3)	Male	5 (4, 5)			
	Female	5 (4, 6)	0.354		
BMI		0.157	0.260		
Group, median (Q1, Q3)	Control	5 (3, 5)	0.332		
	Mild	5 (4, 5)			
	Moderate	4 (4, 5)			
	Severe	5 (4, 6)			

**Univariable analysis:** Spearman correlation was used for age and BMI, Mann-Whitney U test and Kruskal Wallis test were used for gender and group comparison; **Multivariable analysis** = Linear regression analysis

**BMI** = Body Mass Index; **BWT** = Bladder Wall Thickness

There was no significant difference between IPSS-QoL scores of the groups.

A positive statistically significant relationship was reported between the ESS scores and OABSS, as evidenced by that ESS increased, OABSS increased, at the 32.8% level (r=0.328; p=0.045; p<0.01). There was a positive statistically significant relationship between the ESS scores and IPSS-QoL as evidenced by that ESS increased, IPSS-QoL increased, at the 48.3% level (r=0.483; p=0.019; p<0.01).

There was a significant positive relationship between BWT values and OABSS as evidenced by increased BWT and increased OABSS at the level of 48.1% (r=0.481; p=0.002; p<0.01) in the patient group (Table-4).

**Table 4:** Relation of OABSS, IPSS-QoL scores and BWT with age, AHI, Min.SO_2_, Time SO_2_<90% and ESS in patients with OSAS.

		PatientGroup		
		OABSS	IPSS-QoL (n=30)	BWT (mm)
Age (year)	r	0.237	-0.015	0.133
	p	0.164	0.947	0.438
BMI (kg/m^2^)	r	0.222	-0.024	0.149
	p	0.180	0.912	0.371
AHI(/h)	r	0.144	-0.040	0.180
	p	0.388	0.857	0.279
Min.SO_2_	r	-0.258	-0.105	-0.074
	p	0.117	0.634	0.659
Time SO_2_ <90 (%)	r	0.263	0.127	0.150
	p	0.111	0.564	0.370
ESS	r	0.328	0.483	0.208
	**p**	**0.045** *	**0.019** *	**0.210**
BWT(mm)	r	0.481	0.285	1.000
	p	0.002^**^	0.188	-

**r** = Spearman Correlation Coefficient;

* p<0.05

**Abbreviations: BMI** = Body Mass Index; **AHI** = Apne Hypopne Index; **Min.SO_2_** = Minimun oxygen saturation; **Time SO_2_ <90** = The time spent below 90% of oxygen saturation; **ESS** = Epworth Sleepiness Scale; **BWT** = Bladder Wall Thickness; **OABSS** = Overactive Bladder Symptom Score; **IPSS-QoL** = International Prostate Symptoms Score; **BWT** = Bladder Wall Thickness

Multivariate analysis evaluating the effects of age, gender, BMI and groups on nocturia and urgency showed no significant difference. In multivariate analysis, effects of each variable including age, gender, and BMI on UI were significant (χ^2^=19.491, p=0.003). It was found that the rate of UI was 11.969-fold higher in female cases [Exp (Beta) (95% CI): 11.969 (1.236, 115.898), p=0.039].

The Min.SO_2_ value of patients with urinary urgency in the patient group was found to be significantly lower (p=0.014; p<0.05). The time spent <90% SO_2_ was significantly longer in patients with urgency (p=0.009; p<0.01).

## DISCUSSION

In our study, the OABSS of OSAS patients were found to be significantly higher than those of the control group. Overactive bladder syndrome is defined as a symptom-based clinical diagnosis of urinary urgency, with or without urge incontinence, usually with urinary frequency and nocturia, in the absence of infections or other evident pathological features (5). Several studies have been conducted to estimate the incidence of nocturia (12) and erectile dysfunction in patients with OSAS (13). However, the co-occurrence of OAB with OSAS has been only recently demonstrated. In a study conducted in 2009 by Kemmer et al. (14), the prevalence of OAB was reported to be 39% in male patients with OSAS. This rate was found to be higher than the prevalence rate reported for male population in Europe. In the same study, OABSS was found to be significantly higher in male patients with mild and moderate OSAS when compared to patients with mild OSAS and upper airway resistance syndrome (UARS).

In our study, we found no correlation between OABSS and age, AHI, Min.SO_2_, and time spent <90% SO_2_. In our study, we found a significant positive relationship between OABSS and ESS measurements in the patient group. We identified a significant correlation between OABSS and female gender. We also demonstrated that UI was also higher in female patients. Small number of subjects in the control group is definitely a limitation of the present study. However, in European studies, prevalence of OAB was reported to be 4-15% in men and 14-40% in women (7).

In a study conducted by Ipekci et al. (15) in 2016, no significant difference was also found between the severity of OSAS and the prevalence of OAB in 140 female patients with OSAS. In another study, OAB symptom scores were higher in men with moderate to severe OSAS compared to men with mild OSAS (14). In our study, Min. SO_2_ measurements were significantly lower in patients that were found to have urgency problems in the patient group, whereas the time spent <90% SO_2_ was significantly higher. Overactive bladder syndrome induced by nerve destruction has been reported in patients with diabetes (16) and stroke (17). Thus, it is tempting to speculate that the mechanism of OSAS induced-OAB may also be related to hypoxia (14). Patients with OSAS experience numerous repetitive apneic events during sleep, caused by partial or complete upper airway obstruction. The results show intermittent hypoxia and hypercapnia. In the study conducted by P. Lüdemann et al. (18) in 2001, recurrent intermittent hypoxemia was shown to be an independent risk factor for axonal damage of peripheral nerves. We demonstrated a correlation between urgency and hypoxia, although we were unable to find any relationship between OAB syndrome and hypoxia. We suggest that hypoxia-induced ischemic nerve damage in OSAS may lead to urgency problems. In a study conducted by Ipekci et al. (15) in 2016, 140 female patients with moderate to severe OSAS showed improvement in the OABSS with continuous positive airway pressure (CPAP) treatment (15).

We did not observe any difference in BWT measurements between the patient and control groups. In patients with OAB, BWT measurements are usually performed with transvaginal US in women, and consistent results have been reported (9). In a study conducted by Abou-Gamrah A et al. (19) in 2014, BWT measurement using transvaginal US was reported to be an effective diagnostic tool to predict OAB in women presenting with lower urinary tract symptoms. However, in a study conducted in 2016, no relationship was reported between OAB and BWT in 687 women with OAB, as assessed by transvaginal US (20). In a review conducted by Oelke et al. in 2013, transvaginal ultrasound measurement of BWT was reported as a reliable method of diagnosing detrusor overactivity (DO) in women with lower urinary tract symptoms (LUTS), and BWT decreases during antimuscarinic therapy. This review compared transvaginal, translabial/transperineal and suprapubic approaches for ultrasound BWT/detrusor wall thickness (DWT) measurement. Few data were available on the use of translabial or suprapubic ultrasound in women (21). In a study by Uçer et al. in 2016 (10) where 38 women with OAB were evaluated with transabdominal US, pre- and post-voiding BWT measurements were found to be significantly higher in patients with OAB when compared to the healthy control group. The authors considered transabdominal US to be more applicable than transvaginal US and also more preferable for female outpatients in daily practice of urology. In another study, the use of routine transabdominal US was recommended by the authors, despite the superiority of transvaginal US in pelvis evaluation (22). In our search for literature studies, we did not identify any study on BWT measurement in patients with OSAS. We performed BWT measurements using transabdominal US. No difference was found between the patient and control groups in terms of BWT measurements. However, in the patient group, we found a significant positive correlation between BWT measurements and OAB scores.

In our study, we did not demonstrate any significant difference in the IPSS-QoL scores between male subjects in the patient and the control groups. In a study by Kemmer et al. in 2009 (14) that included 85 participants, no difference in IPSS-QoL was reported between the OSAS and UARS groups (14).

The authors consider that the present study is worthy as it is the first study to evaluate the relationship between OSAS and BWT. Small number of subjects in the patient group and the control group is a limitation of the study. We did not find any difference in BWT measurements between the patient and the control groups. However, OAB symptom scores were higher in patients with OSAS compared with the control group and there was a positive correlation between OABSS and BWT measurements in patients with OSAS. The authors therefore suggest that weak correlation between OSAS and BWT measurements can be detected by increasing the number of control subjects.

## CONCLUSIONS

In conclusion, OSAS is associated with OAB syndrome. Urgency, which is considered to be a key symptom of OAB, is also known to be associated with hypoxia in patients with OSAS. These findings have clinical implications, as untreated OAB and urgency may cause a relevant attenuation in quality of life of affected patients. Therefore, OAB should be investigated in all patients with OSAS.

The present study observed no difference in BWT measurements between the patients and the control group. However, the present study found a correlation between BWT measurements and OAB scores in patients with OSAS. Further studies are required to gain a better understanding of possible use of BWT measurements for OAB in patients with OSAS.
